# Macrophage depletion reduced brain injury following middle cerebral artery occlusion in mice

**DOI:** 10.1186/s12974-016-0504-z

**Published:** 2016-02-13

**Authors:** Yuanyuan Ma, Yaning Li, Lu Jiang, Liping Wang, Zhen Jiang, Yongting Wang, Zhijun Zhang, Guo-Yuan Yang

**Affiliations:** Department of Neurology, Ruijin Hospital, School of Medicine, Shanghai Jiao Tong University, Shanghai, 200025 China; Neuroscience and Neuroengineering Research Center, Med-X Research Institute and School of Biomedical Engineering, Shanghai Jiao Tong University, 1954 Hua Shan Road, Shanghai, 200030 China

**Keywords:** Brain, Ischemia, Macrophage, Microglia, Myelin

## Abstract

**Background:**

Macrophages are involved in demyelination in many brain diseases. However, the role of macrophages in the recovery phase of the ischemic brain is unknown. The present study aims to explore the role of macrophages in the ischemic brain injury and tissue repair following a 90-min transient middle cerebral artery occlusion in mice.

**Methods:**

Clodronate liposomes were injected into mice to deplete periphery macrophages. These mice subsequently underwent middle cerebral artery occlusion. F4/80^+^ and CD68^+^ cells were examined in the mouse spleen and brain to confirm macrophage depletion at 14 days after middle cerebral artery occlusion. Modified neurological severity scores were used to evaluate the behavioral function between 1 and 14 days after middle cerebral artery occlusion. MBP, Iba1, and CD31 immunostaining were performed to determine myelin lesion, microglia activation, and microvessel density.

**Results:**

Clodronate liposomes depleted 80 % of the macrophages in the mouse spleen and reduced macrophage infiltration in the mouse brain. Macrophage depletion reduced the myelin damage in the ipsilateral striatum and microglia activation in both the ipsilateral cortex and striatum, enhanced the microvessel density in the peri-infarct region, attenuated brain atrophy, and promoted neurological recovery following middle cerebral artery occlusion.

**Conclusions:**

Our results suggested that macrophage depletion is a potential intervention that can promote tissue repair and remodeling after brain ischemia, reduce demyelination and microglia activation, and enhance focal microvessel density.

**Electronic supplementary material:**

The online version of this article (doi:10.1186/s12974-016-0504-z) contains supplementary material, which is available to authorized users.

## Background

Ischemic stroke is the second leading cause of death and the most common cause of disability worldwide [1, 2]. There still lacks effective therapy except for timely endovascular treatment and thrombolysis of ischemic stroke onset [3, 4]. However, only 4–7 % of patients with acute ischemic stroke are eligible for thrombolytic treatment due to the narrow therapeutic time window and the risk of hemorrhagic transformation [5]. It remains critical to develop new strategies to reduce brain injury and promote neurological function recovery after ischemic stroke [6].

Ischemic stroke is caused by severe stenosis or occlusion of a cerebral artery. It can subsequently lead to evere neural injury, neuronal necrosis, and apoptosis. Necrotic or apoptotic tissues release a variety of pro-inflammatory cytokines and damage-associated molecular patterns (DAMPs), which activate brain resident microglia and recruit peripheral immune cells into the brain. Macrophages and neutrophils are the key players in ischemia-induced inflammatory response [7]. However, there are different infusion patterns among macrophages, neutrophils, and other immune cells in mice following middle cerebral artery occlusion (MCAO). Periphery macrophages infiltrate into the brain as early as 12 h and recruit more neutrophils into the ischemic brain by releasing pro-inflammatory cytokines and up-regulating adhesion molecules in endothelial cells [8, 9]. The infiltration of neutrophils and lymphocytes is mostly limited at the early stage of ischemic stroke while the infiltration of periphery macrophages persists through the recovery phase of ischemic stroke [10]. The role of periphery macrophages during ischemic stroke is under debate. On the one hand, macrophage infiltration was thought to exacerbate focal inflammatory response and further damage the brain [11–13]. Macrophages can specifically bind with myelin oligodendrocyte glycoprotein (MOG) and infiltrate into the ischemic brain, increase brain infarct volume, and exacerbate neurological deficit after 4 days of transient MCAO in mice [14]. On the other hand, macrophages also participate in phagocytosis of necrotic debris at 3 and 6 days after transient MCAO [15]. Macrophage infiltration at the early stage of tissue injury is critical for tissue repair and remodeling but is not as important during the late phase of tissue recovery [16, 17]. In a mouse model of spinal cord injury, periphery macrophages contributed to fibrotic scar formation and inhibited axonal growth at the recovery phase of spinal cord injury [18]. Based on these reported observations, we hypothesized that the infiltration of periphery macrophages possibly is detrimental for tissue repair at the recovery phase of ischemic stroke.

To directly explore the role of periphery macrophages during the recovery phase of ischemic stroke, we depleted the periphery macrophages by intraperitoneally injecting clodronate liposomes into mice at 1 day before and 4 and 9 days after transient MCAO. This strategy ensures persistent macrophage depletion during the whole period of ischemic brain injury [17–19]. We aim to explore (1) whether macrophage depletion facilitates the neurological functional recovery during the recovery phase after ischemic stroke, (2) whether macrophage depletion reduces myelin lesion, (3) whether macrophage depletion affects brain resident microglia response, and (4) whether macrophage depletion enhances the microvessel density.

## Methods

### Transient MCAO in mice

**Fig. 1:**
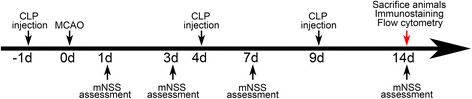
A diagram of the experimental design. Mice were treated with clodronate liposomes 1 day before transient MCAO. Neurological functions were assessed using modified neurological severity scores following 1, 3, 7, and 14 days after transient MCAO. The mouse brain was collected for the immunohistochemistry, and the spleens were obtained for flow cytometry at 14 days after MCAO

**Fig. 2:**
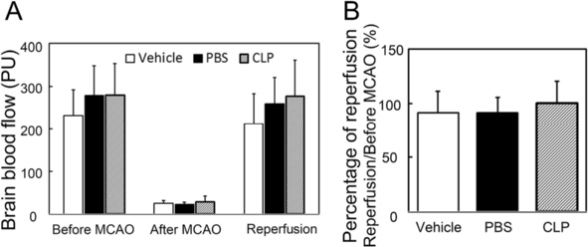
Brain blood flow in mice during MCAO. **a** Representation of brain blood flow before MCAO (as a baseline), after MCAO (when the cerebral artery was occluded), and reperfusion (when the suture was withdrawn from the cerebral artery). **b** Percentage of brain blood flow reperfusion (reperfusion/before MCAO) among groups. There was no significance on the percentage of brain blood flow reperfusion among groups. *n* = 11–13 per group

All animal procedures were carried out in accordance with the guideline of the Institutional Animal Care and Use Committee (IACUC) of Shanghai Jiao Tong University, Shanghai, China. The experimental protocol is illustrated in Fig. 1. Forty-four adult male Institute of Cancer Research (ICR) mice (Sippr-BK, Shanghai, China) weighing 25–30 g were used in the study. Transient MCAO surgery was performed as described previously [20]. Briefly, mice were anesthetized with ketamine/xylazine (100 mg/10 mg/kg, Sigma, St. Louis, MO). After making an incision at the midline of the neck, the left common carotid artery, external carotid artery, and internal carotid artery were isolated. A 6-0 silica-coated nylon suture was gently inserted through the external carotid artery and advanced along the internal carotid artery and stopped at the origin of middle cerebral artery. After 90 min of ischemia, the suture was withdrawn and reperfusion was achieved. The successful ischemia and reperfusion were validated by monitoring the changes of cerebral blood flow using a laser Doppler flowmetry (Moor Instruments, Axminster, Devon, UK). Animals were included into further characterizations when the brain blood flow decreased for at least 80 % compared to the baseline and returned to 80 % of the baseline after suture withdrawal (Fig. 2). The mortality rates of animals following MCAO for the control liposome (Vehicle), phosphate-buffered saline (PBS), and clodronate liposome (CLP) groups were 21.4, 14.3, and 18.8 %, respectively. There was no significant difference of the mortality among the groups.

### Neurobehavioral examination

Neurobehavioral tests were carried out by two investigators who were blinded to the experimental groups using a modified neurological severity score (mNSS) system. mNSS ranges from 0 to 14, in which 0 represents normal and 14 represents the highest degree of neurological deficiency [21]. mNSS is a comprehensive assessment of neurological function including motor, sensory, balance, and reflex tests. For the motor test, the bend and torsion of limbs were observed by holding up the tail of the mouse (0–3). The posture of walking on the floor was also assessed (0–3). For the balance test, the mice were placed on a beam. The neurological deficiency was assessed according to whether the mouse could keep balance on the beam, limbs fall off the beam, and walk through the beam (0–6). For the sensory and reflex tests, pinna and corneal reflex were examined, respectively (0–2). The higher the scores, the more severe is the injury.

### The measurement of brain atrophy volume

The mice were sacrificed following 14 days of transient MCAO and perfused with 0.9 % saline followed by 4 % paraformaldehyde. The brain samples were removed and post-fixed in 4 % paraformaldehyde for 6 h and dehydrated in 30 % sucrose until the brain sank into the bottom of a 15-ml tube. Then the brains were frozen in −80 °C for 2 days and cut into 20-μm sections from the anterior commissure to the hippocampus. A total of 20 coronal sections were mounted on the slide and used for cresyl violet staining. The distance between adjacent sections was 200 μm. The other sections used for immunohistochemistry were collected in the anti-freezing solution and stored at −20 °C until use. The atrophy area was measured using the ImageJ software (National Institutes of Health, Bethesda, MD). The atrophy volume was calculated with the formula *V* = ${\displaystyle {\sum}_1^n\left[\left({\mathrm{S}}_n+\sqrt{{\mathrm{S}}_n*{\mathrm{S}}_{n+1}}+{\mathrm{S}}_{n+1}\right)*\frac{h}{3}\right]}$, in which *h* was the distance between two adjacent sections and S_*n*_ and S_*n* + 1_ were the atrophy areas of two adjacent sections [22, 23].

### Macrophage depletion and clodronate liposome administration

Clodronate liposomes were widely used to deplete peripheral macrophages [24, 25]. Clodronate was encapsulated in liposomes with a concentration of 7 mg clodronate per milliliter, a concentration that can ensure the depletion of 90 % of macrophages within 24 to 36 h after systemic administration [26]. Clodronate liposomes (F70101C-A, FormuMax Scientific, Inc., Palo Alto, CA) were intraperitoneally injected at 1 day before and 4 and 9 days after transient MCAO. Control liposomes (F70101C-A, FormuMax Scientific, Inc., Palo Alto, CA) and PBS were used as the controls. The dose of clodronate liposomes was adjusted according to the manufacturer’s instructions (0.2 ml/20–25 g). The efficiency of macrophage depletion was verified at 14 days after MCAO by examining the number of F4/80^+^ macrophages in the spleen and CD68^+^ macrophages in the brain by flow cytometry and immunostaining.

### Spleen cell isolation and flow cytometry

Flow cytometry was performed on the spleen samples collected on day 14 after MCAO. The mice were deeply anesthetized and the spleens were removed and collected in a 1.5-ml tube. To obtain single cell suspension, the spleens were grinded in PBS using a mechanical trituration method through a 100-μm cell strainer (Corning, New York, NY). The cell suspension was centrifuged for 5 min at 1000 rpm at 25 °C, and the supernatant was discarded. Red blood cells were removed by re-suspending the cells in red blood cell lysis buffer (NH4Cl 8.29 g, KHCO3 1 g, EDTA-2Na 37.2 mg, diluted in distilled water, at pH = 7.4) for 30 s. Then the cell suspension was centrifuged for 5 min at 1000 rpm at 25 °C again and the supernatant was discarded. After washing in PBS, the cells were re-suspended in 1 ml PBS. Single cell suspension of 200 μl was incubated with fluorescence-conjugated rat–anti-mouse F4/80-FITC (1:200, BioLegend, San Diego, CA) for 30 min at 4 °C. Then the cells were centrifuged for 5 min at 2500 rpm at 25 °C. After discarding the supernatant, the cells were washed once with PBS. Then the cells were re-suspended in 300 μl PBS and analyzed by a flow cytometry (BD Biosciences, San Jose, CA). A minimum of 10,000 events were acquired for each sample.

### Western blot

The ischemic striatum was collected and sonicated in the protein lysis buffer (RIPA with protease cocktail inhibitor, phosphatase inhibitor). The brain homogenate was centrifuged at 12,000 rpm and the supernatant was collected. Protein concentrations were determined with a BCA kit (Thermo Scientific, Waltham, UK). Equal amounts of protein were loaded onto 15 % resolving gel for electrophoresis. The proteins were transferred onto a nitrocellulose membrane (Whatman Inc., Florham Park, NJ) and incubated with the primary antibodies of MBP (1:1000 dilution, Abcam, Cambridge, MA) and β-actin (1:1000 dilution, Abcam, Cambridge, MA) at 4 °C overnight. After washing three times using a TBST buffer, the membrane was incubated with horseradish peroxidase (HRP)-conjugated anti-rat or anti-mouse immunoglobulin G for 1 h at room temperature, washed, and then reacted with an enhanced chemiluminescence substrate (Pierce, Rockford, IL). The result of chemiluminescence was semi-quantified using the ImageJ software (National Institutes of Health, Bethesda, MD).

### Immunohistochemistry

Myelin basic protein (MBP) immunostaining was performed following DAB immunostaining protocol (Vector Labs, Burlingame, CA) as previously described with minor modifications [20]. Briefly, the brain sections were incubated with 0.3 % H_2_O_2_ for 30 min at room temperature. After rinsing with PBS, 0.3 % Triton X-100 was added for 10 min, then the coronal sections were incubated with horse serum for 1 h at room temperature. The brain sections were then incubated in a primary antibody of MBP (1:500, Abcam, Cambridge, MA) at 4 °C overnight. After rinsing with PBS for three times, the brain sections were incubated with a biotinylated secondary antibody and Vectastain ABC solution (Vector Labs, Burlingame, CA) for 1 h. The brain sections were then examined under an optical microscope. Three fields in the peri-infarct region of the ipsilateral striatum and the corresponding contralateral striatum were imaged in each section. A total of five sections (taking every other section spaced 200 μm apart) were evaluated for each mouse. The mean integrated optical density (IOD) was measured by the ImageJ Pro Plus 6.0 software (Media Cybernetics, Bethesda, MO). The ratio of mean IOD between the ipsilateral and contralateral was used for further analysis.

For macrophage, microglia, and microvessel immunostaining, the brain sections were incubated in the primary antibodies of CD68 (1:200, AbD Serotec, Kidlington, UK), Iba1 (1:200, WAKO, Osaka, Japan), and CD31 (1:150, R&D, Minneapolis, MN) at 4 °C overnight. After rinsing with PBS for three times, the brain sections were incubated with the secondary antibodies: Alexa Fluor 488-conjugated donkey anti-rat, Alexa Fluor 488-conjugated donkey anti-rabbit, or Alexa Fluor 594-conjugated donkey anti-goat (1:500, Invitrogen, Carlsbad, CA) for 1 h at room temperature. Then the brain sections were rinsed with PBS for three times and incubated with 4′, 6-diamidino-2-phenylindole (DAPI, Life Technologies, Mulgrave, VIC, Australia) for 5 min at room temperature. After rinsing with PBS, the brain sections were covered and sealed with mounting medium (Vector Labs, Burlingame, CA) for further study. Negative controls (without primary antibodies) were included in each immunohistochemistry experiment. Additional figures show this in more detail (see Additional file 1).

### Cell and vessel counting

The brain tissues were visualized using a confocal microscope (Leica, Solms, Germany). For Iba1^+^ microglia counting, the field in the peri-infarct region of the cortex and striatum was imaged per section. For CD68^+^ macrophages and CD31^+^ microvessels, three fields in the peri-infarct region of the striatum were imaged per section. A total of five sections with a 200-μm interval were selected per mouse. The number of positive cells was counted by the investigators who were blinded to the experimental groups. DAPI-stained nuclei were counted by the ImageJ Pro Plus 6.0 software (Media Cybernetics, Bethesda, MO). The average number of positive cells per section and the percentage of the positive cells to total cells stained by DAPI were used for data analysis [27].

### Statistical analysis

Data were presented as mean ± SD. Statistical significance among groups were evaluated by one-way ANOVA followed by the Bonferroni (homogeneity of variance) or the Tamhane test (heterogeneity of variance) using the SPSS 18.0 software (SPSS Inc., Chicago, IL). For non-parametric analysis, the Kruskal–Wallis test and Mann–Whitney *U* test were applied. A value of *p* < 0.05 was considered significant.

## Results

### Macrophages were successfully depleted by clodronate liposome treatment

**Fig. 3:**
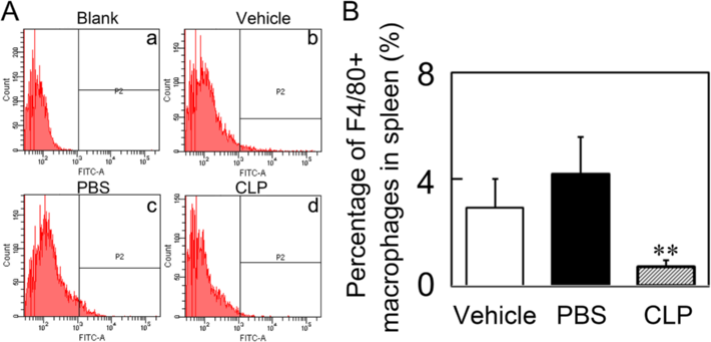
Clodronate liposome treatment depleted macrophages in the spleen. **a** Flow cytometry analysis shows the percentage of F4/80^+^ macrophages in the spleen of Vehicle (**b**), PBS (**c**), and CLP (**d**) groups at14 days after MCAO. **b** Bar graph shows quantification of the percentage of F4/80^+^ macrophages in the spleen of Vehicle, PBS, and CLP groups. Data are mean ± SD, *n* = 5–8 per group. **p < 0.01, CLP vs. Vehicle or PBS. CLP = clodronate liposomes, PBS = phosphate-buffered saline, Vehicle = control liposomes

To deplete periphery macrophages during transient MCAO, the mice were subjected to clodronate liposome injection. At 14 days after injection, 80 % of F4/80^+^ macrophages were depleted in the spleen in the clodronate liposome-treated mice compared to the control mice (Fig. 3, *p <* 0.05), this result suggested the success of macrophage depletion. Animals that received clodronate liposomes showed no visible disorders such as infection, reduced appetite, or inhibition of motor activity.

### Macrophage infiltration was reduced in the brain of MCAO after clodronate liposome injection

**Fig. 4:**
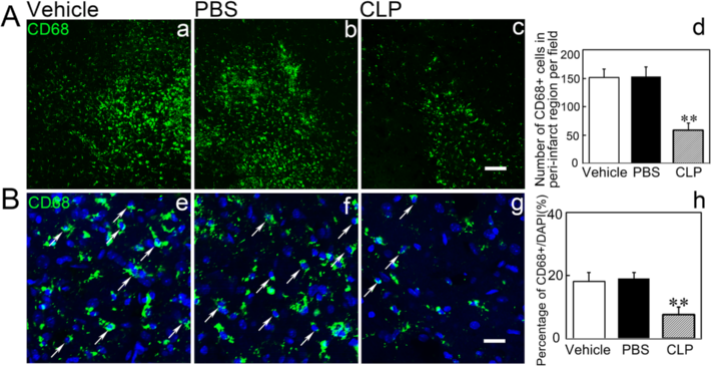
Clodronate liposome treatment reduced macrophage infiltration in the brain after MCAO. **a** Representative images of CD68^+^ macrophages (*green*) of the ipsilateral striatum in Vehicle (*a*), PBS (*b*), and CLP (*c*) groups at 14 days after MCAO. *Scale bar* = 100 μm. Quantification of the number of CD68^+^ macrophages in the peri-infarct region of the ipsilateral striatum (*d*). **b** Representative images of CD68^+^ macrophages (*green*) and DAPI-stained cells (*blue*) of the ipsilateral striatum in Vehicle (*e*), PBS (*f*), and CLP (*g*) groups at 14 days after MCAO. *Scale bar* = 20 μm. Quantification of the percentage of CD68^+^/DAPI cells in the peri-infarct region of the ipsilateral striatum (*h*). Data are mean ± SD, *n* = 4 per group. **p < 0.01, CLP vs. Vehicle or PBS. CLP = clodronate liposomes, PBS = phosphate-buffered saline, Vehicle = control liposomes

To further clarify whether periphery macrophage depletion could reduce macrophage infiltration in the brain, we examined the number of CD68^+^ macrophages in the peri-infarct region of the ipsilateral striatum. We found that macrophages were reduced in the clodronate liposome-treated mice compared to the control mice (Fig. 4, *p* < 0.05); this result indicated that periphery macrophage depletion could reduce macrophage infiltration in the brain after ischemia.

### Macrophage depletion reduced the brain atrophy volume and neurological deficits in MCAO mice

**Fig. 5:**
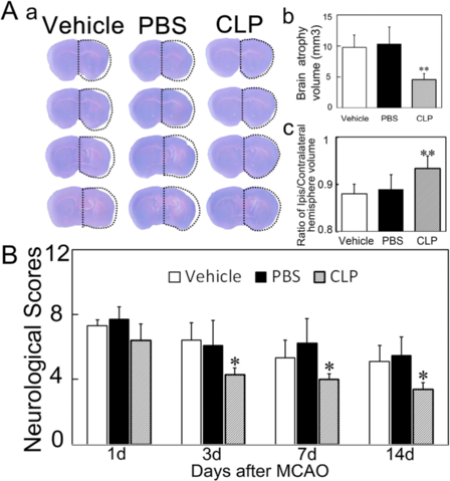
Macrophage depletion improved neurological outcomes in mice following transient MCAO. **a** A series of cresyl violet stained coronal sections represented the brain atrophy in CLP, PBS, and Vehicle groups at 14 days after MCAO (**a**). Quantification of the brain atrophy volume is presented by the brain volume of contralateral subtracted ipsilateral hemisphere at 14 days after MCAO (*b*). Quantification of brain volume presented by ratio of ipsilateral/contralateral hemisphere brain volume at 14 days after MCAO (*c*). Data are mean ± SD, *n* = 7–8 per group. **p < 0.01, CLP vs. Vehicle or PBS. **b** Bar graph shows the mNSS assessment in CLP, PBS, and Vehicle groups at 1, 3, 7, and 14 days after MCAO. Data are mean ± SD, *n* = 5–8 per group. *p < 0.05, CLP vs. Vehicle or PBS. mNSS = modified neurological severity scores

To analyze the influence of macrophage depletion on tissue repair, we measured the brain atrophy volume at 14 days after MCAO. The result showed that the brain atrophy volume was much smaller in the clodronate liposome-treated mice than that in the control mice (Fig. 5a, *p <* 0.05). To examine the role of macrophage depletion on neurological outcomes, we assessed the neurological deficits using the mNSS system at 1, 3, 7, and 14 days after MCAO. The results showed that neurological scores were lower at 3 days and maintained at low level for 14 days after MCAO in the clodronate liposome-treated mice compared to the control mice (Fig. 5b, *p <* 0.05).

### Macrophage depletion reduced myelin lesion in ischemic mice

**Fig. 6:**
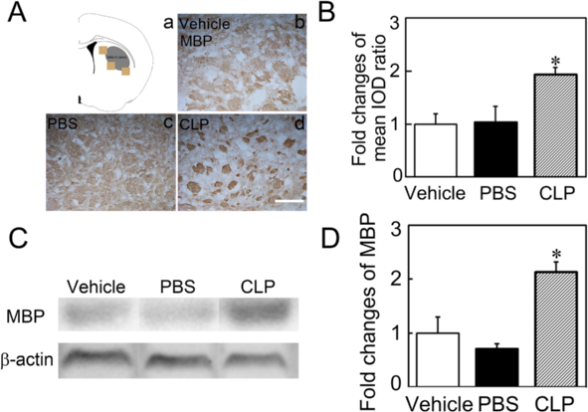
Macrophage depletion reduced myelin lesion. **a** *Light brown boxes* show the interested areas in the ipsilateral striatum (*a*). Photomicrographs show MBP-stained myelin in the peri-infarct region of the ischemic striatum in Vehicle (*b*), PBS (*c*), and CLP (*d*) groups at 14 days after MCAO. *Scale bar* = 100 μm. **b** Bar graph represents quantification of fold changes of mean IOD ratio (ipsilateral/contralateral hemisphere) in the CLP group compared to the PBS or Vehicle group. Data are mean ± SD, *n* = 4 per group. *p < 0.05, CLP vs. Vehicle or PBS. IOD = integrated optical density. **c** MBP expression in the ischemic striatum was determined by western blot. **d** Quantification of MBP expression by normalizing to the β-actin level in each group and set the Vehicle treated group as 1. Data are mean ± SD, *n* = 3 per group. *p < 0.05, CLP vs. Vehicle or PBS

Myelin, a major component of the white matter, is vulnerable to ischemia, and the loss of myelin contributes to impaired neurological outcomes. To understand why macrophage depletion facilitated the recovery of ischemic stroke, we examined MBP expression to assess the myelin lesion using immunostaining and western blot. The results showed that MBP expression was higher in the clodronate liposome-treated mice compared to the control mice (Fig. 6, *p* < 0.05), suggesting that macrophage depletion reduced myelin lesion.

### Macrophage depletion reduced microglia activation both in the cortex and striatum

**Fig. 7:**
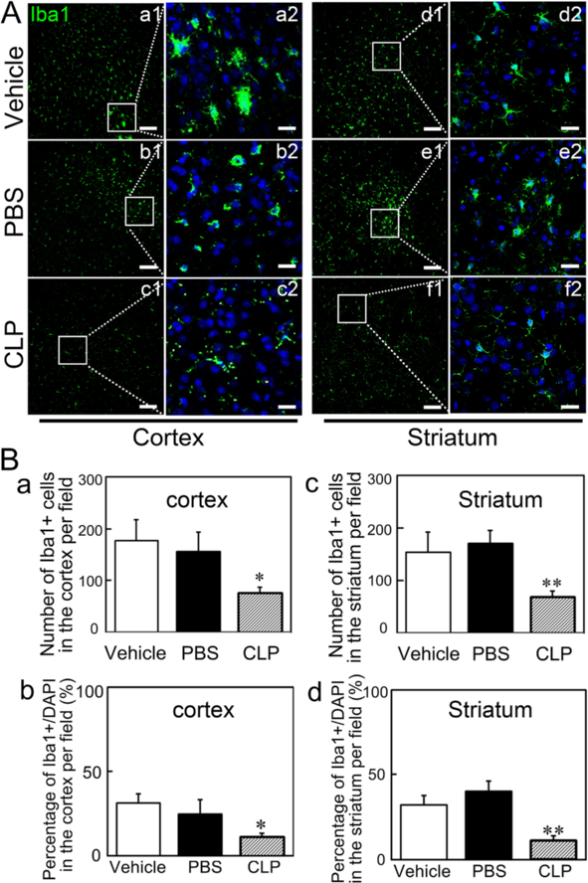
Macrophage depletion reduced microglia activation. **a** Photomicrographs represented Iba1 (*green*)-stained microglia in the cortex and striatum in CLP, PBS, and Vehicle groups at 14 days after MCAO. *Right panels* at each column (*a2 to f2*) presented a higher magnification of the images of *left panels* labeled by the *boxes* (*a1 to f1*). *Scale bar* = 100 μm in *a1* to *f1* and 20 μm in *a2* to *f2*. **b** Quantification of the number of Iba1^+^ microglia located in the cortex (**a**) and striatum (**c**) and the percentage of Iba1^+^ /DAPI cells located in the cortex (*b*) and striatum (*d*) at 14 days after MCAO. Data are mean ± SD, *n* = 4 per group. *p < 0.05, **p < 0.01, CLP vs. Vehicle or PBS

Brain resident microglia activation participates in myelin lesion induced by ischemia, and periphery macrophage infiltration might contribute to microglia activation [10]. To further explore the effect of macrophage depletion on microglia activation, we examined microglia activation by Iba1 immunostaining. We found that microglia displayed a classical ramified shape, with long and thin processes in the cortex and striatum in the macrophage depletion group (Fig. 7a). Microglia displayed distinct cell morphology with a larger cell body and more processes in the control groups compared to the macrophage depletion group. Furthermore, we found that macrophage depletion significantly reduced the number of microglia both in the ipsilateral cortex and striatum (Fig. 7b, *p* < 0.05). These data suggested that macrophage depletion reduced microglia activation at least at 14 days after MCAO.

### Macrophage depletion enhanced the microvessel density in MCAO mice

**Fig. 8:**
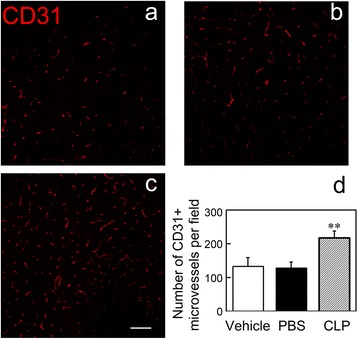
Macrophage depletion enhanced the microvessel density in the peri-infarct region of mice brain after MCAO. Photomicrographs show that CD31^+^ microvessels in the peri-infarct region of the ipsilateral striatum in Vehicle (**a**), PBS (**b**), and CLP (**c**) groups at 14 days after MCAO. *Scale bar* = 100 μm. Bar graph shows the quantification of the number of CD31^+^ cells in the peri-infarct region of the ipsilateral striatum (**d**). Data are mean ± SD, *n* = 4 per group. **p < 0.01, CLP vs. Vehicle or PBS

Macrophages were reported to reduce the microvessel density in diabetic mice, while the microvessel density in the peri-infarct region contributed to recovery after brain injury [22, 23]. Therefore, we explored whether macrophage depletion affected the microvessel density during MCAO. We counted CD31^+^ microvessels in the peri-infarct region of the ipsilateral striatum (Fig. 8a–c). The number of CD31^+^ microvessels increased by 40 % in the macrophage depletion mice compared to the control mice (Fig. 8d, *p* < 0.05). The result suggested that macrophage depletion could increase the microvessel density in the peri-infarct region of the lesion striatum.

## Discussion

In this study, we found that macrophage depletion improved neurological outcomes and promoted tissue repair for 14 days after MCAO. The beneficial outcomes of macrophage depletion correlate with the decrease of myelin loss and microglia activation and the increase of focal microvessel density.

We aimed to explore whether macrophage depletion affects the neurological function recovery at the recovery phase of MCAO. To achieve the maximum efficiency of macrophage depletion, mice were subjected to clodronate liposome injection at 1 day prior to transient MCAO. We gave two additional injections of clodronate liposomes at a 4-day interval until 14 days after transient MCAO [19]. The success of macrophage depletion was verified by quantifying F4/80^+^ macrophages in the spleen at 14 days after MCAO, which showed a reduction of 80 % F4/80^+^ macrophages in the clodronate liposome-treated mice compared to the control mice. Macrophage infiltration was also reduced in the brain after ischemia, which was validated by immunostaining of CD68^+^ macrophages in the brain. Our results showed that persistent macrophage depletion reduced the brain atrophy volume and improved the neurological outcomes at 14 days after MCAO, which suggested that macrophages could impede the neurological function recovery after ischemic stroke.

Both peripheral infiltrated macrophages and brain resident microglia activation were implicated in demyelination-associated diseases such as globoid cell leukodystrophy, multiple sclerosis, spinal cord injury, and traumatic brain injury [28–31]. LPS injection-induced brain inflammation resulted in myelin lesion, which was accompanied by enhanced microglia activation and morphological transition [32]. The number of periphery macrophages correlated with the severity of tissue damage [33]. Macrophage infiltration and brain resident microglia activation contributed to myelin damage via producing pro-inflammatory cytokines, chemokines, reactive oxygen species, and glutamate [34]. Iba1^+^ and CD68^+^ cells infiltrated around the damaged myelin after MCAO, the result suggested that these cells were implicated in ischemia-induced myelin lesion [35]. However, these past studies could not elucidate the specific contribution of brain resident microglia activation and infiltrated macrophages to myelin lesion, because Iba1 and CD68 are markers for both brain resident microglia and peripheral infiltrated macrophages [36, 37]. In our study, we specifically depleted the peripheral macrophages by intraperitoneal injection of clodronate liposomes; thus, the Iba1^+^ cells in the cortex and striatum were dominated by brain resident microglia. There were less Iba1^+^ microglia accumulated in the peri-infarct region of the ipsilateral cortex and striatum in the macrophage depletion mice compared to the control. In addition, macrophage depletion also caused microglia morphological changes in the ipsilateral cortex and striatum in MCAO mice. Microglia displayed a large cell body and thick and short processes in the control brain while displaying small round cell body and ramified morphology in the clodronate liposome-treated mice. These results suggested that peripheral macrophages participated in re-populating microglia in the peri-infarct region after MCAO and contributed to exacerbating myelin damage. The peripheral macrophages could be a potential target for the development of intervention strategies to treat ischemic myelin damage.

Macrophages are critical in promoting angiogenesis by releasing pro-angiogenic factors or supporting the process of microvessel sprouting in tumor [38]. Infiltration of macrophages within 24 h after limb amputation is essential for limb regeneration and tissue recovery via enhancing the microvessel density in the salamanders [25]. To explore whether macrophage depletion affected angiogenesis after ischemic stroke, we examined CD31^+^ microvessels in the peri-infarct region of the striatum at 14 days after ischemia. We found that the number of CD31^+^ cells increased in the peri-infarct region of the ischemic striatum in the macrophage depletion mice compared to the control. This result suggested that macrophage depletion enhanced the microvessel density. Studies demonstrated that macrophage depletion attenuated kidney injury by up-regulating stromal cell-derived factor-1 (SDF-1) expression [39]. SDF-1 could enhance pro-angiogenic factors such as CCL and vascular endothelial growth factor (VEGF) expression and promote angiogenesis under different pathological conditions [22, 40]. Therefore, one possible explanation for the macrophage depletion to enhance the microvessel density is possibly the up-regulation of pro-angiogenic factors such as VEGF or chemokines such as SDF-1 in the brain. Furthermore, macrophage depletion may improve neurological function via promoting bone marrow stem cell mobilization to the ischemic brain [41]. The relationship of macrophages and the mobilization of bone marrow stem cells during ischemic stroke awaits further investigation.

## Conclusions

Although macrophage depletion prior to ischemic stroke onset is inappropriate for clinical application, it is conducted to explore the role of macrophages during ischemic stroke. Our results showed that macrophage depletion is beneficial for reducing brain injury and promoting tissue recovery following 14 days of ischemic stroke. However, the mechanism by which macrophage depletion improved recovery after ischemia needs to be further investigated. In conclusion, we explored the role of macrophages at the recovery phase of ischemic stroke via peripheral macrophage depletion induced by clodronate liposomes. Our results showed that macrophage depletion reduced the brain atrophy volume and improved neurological outcomes after MCAO. Alleviation of ischemic brain injury induced by macrophage depletion may be due to the reduced myelin lesion and microglia activation and the enhanced microvessel density. Our study suggested that temporary inhibition of peripheral macrophages could be a promising intervention strategy for the ischemic stroke therapy.

## Additional file

Additional file 1
**Supplementary figures and figure legends.** (437 kb)

## Abbreviations

CLP: clodronate liposomes
DAMPs: damage-associated molecular patterns
DAPI: 4′, 6-diamidino-2-phenylindole
HRP: horseradish peroxidase
IACUC: institutional animal care and use committee
ICR: institute of cancer research
IOD: integrated optical density
MBP: myelin basic protein
MCAO: middle cerebral artery occlusion
mNSS: modified neurological severity scores
MOG: myelin oligodendrocyte glycoprotein
PBS: phosphate-buffered saline
SDF-1: stromal cell-derived factor-1
Vehicle: control liposomes
