# Supplementary material for: Macrophage depletion reduced brain injury following middle cerebral artery occlusion in mice
Source: J Neuroinflammation. 2016 Feb 13;13:38. doi: 10.1186/s12974-016-0504-z (PMC4752808; doi:10.1186/s12974-016-0504-z)
Supplement: Additional file 1 — Supplementary figures and figure legends. (437 kb) [file 12974_2016_504_MOESM1_ESM.pdf]

Supplementary figures and figure legends

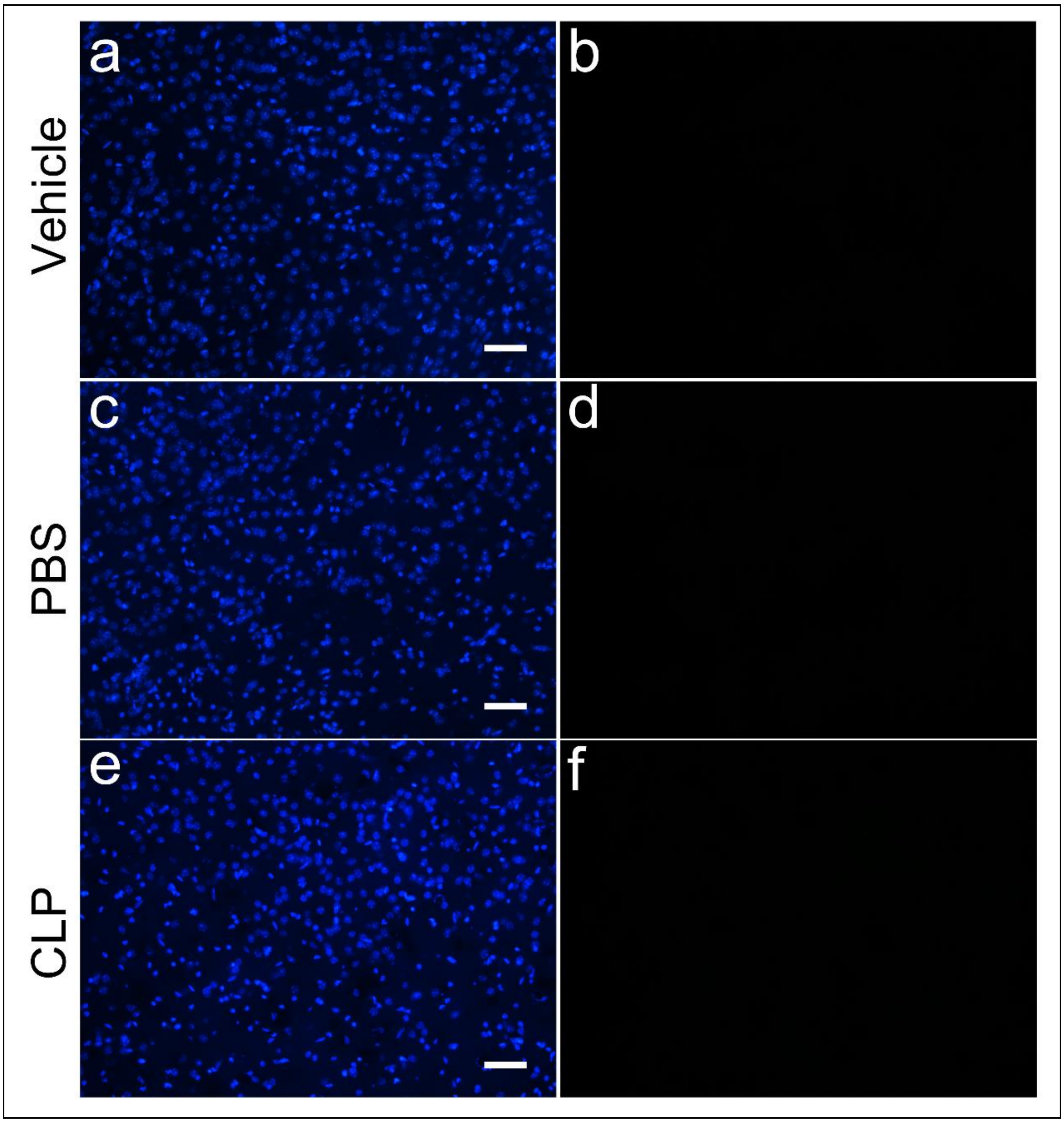

Supple 1. Negative control of CD68 immunostaining (no primary antibody of CD68). Presentation of DAPI stained nuclei (**a, c, e,**) and CD68 negative immunostaining (**b, d, f**) in the peri-infarct region of the striatum of Vehicle, PBS, and CLP treated group. Scar bar=50 μm.

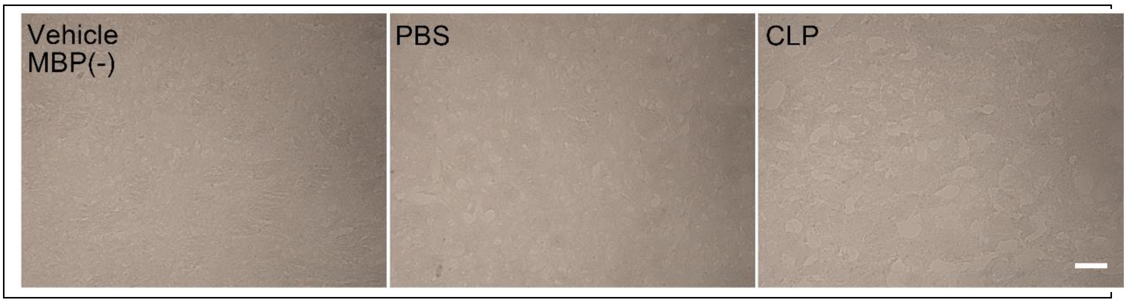

Supple 2. Negative control of MBP immunostaining (no primary antibody of MBP). Presentation of MBP negative immunostaining in the peri-infarct region of the striatum of Vehicle, PBS, and CLP treated group. Scar bar=50 μm.

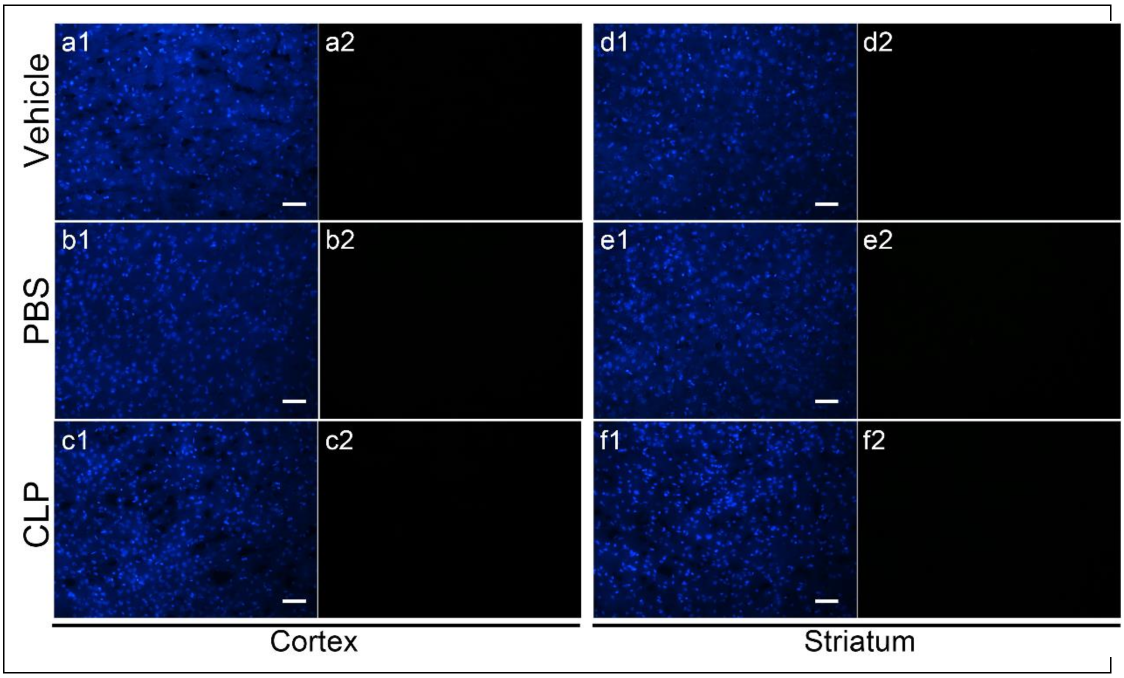

Supple 3. Negative control of Iba1 immunostaining (no primary antibody of Iba1). Presentation of DAPI stained nuclei (**a1-f1**) and Iba1 negative immunostaining (**b2-f2**) in the peri-infarct region of the cortex and striatum of Vehicle, PBS, and CLP treated group. Scar bar=50 μm.

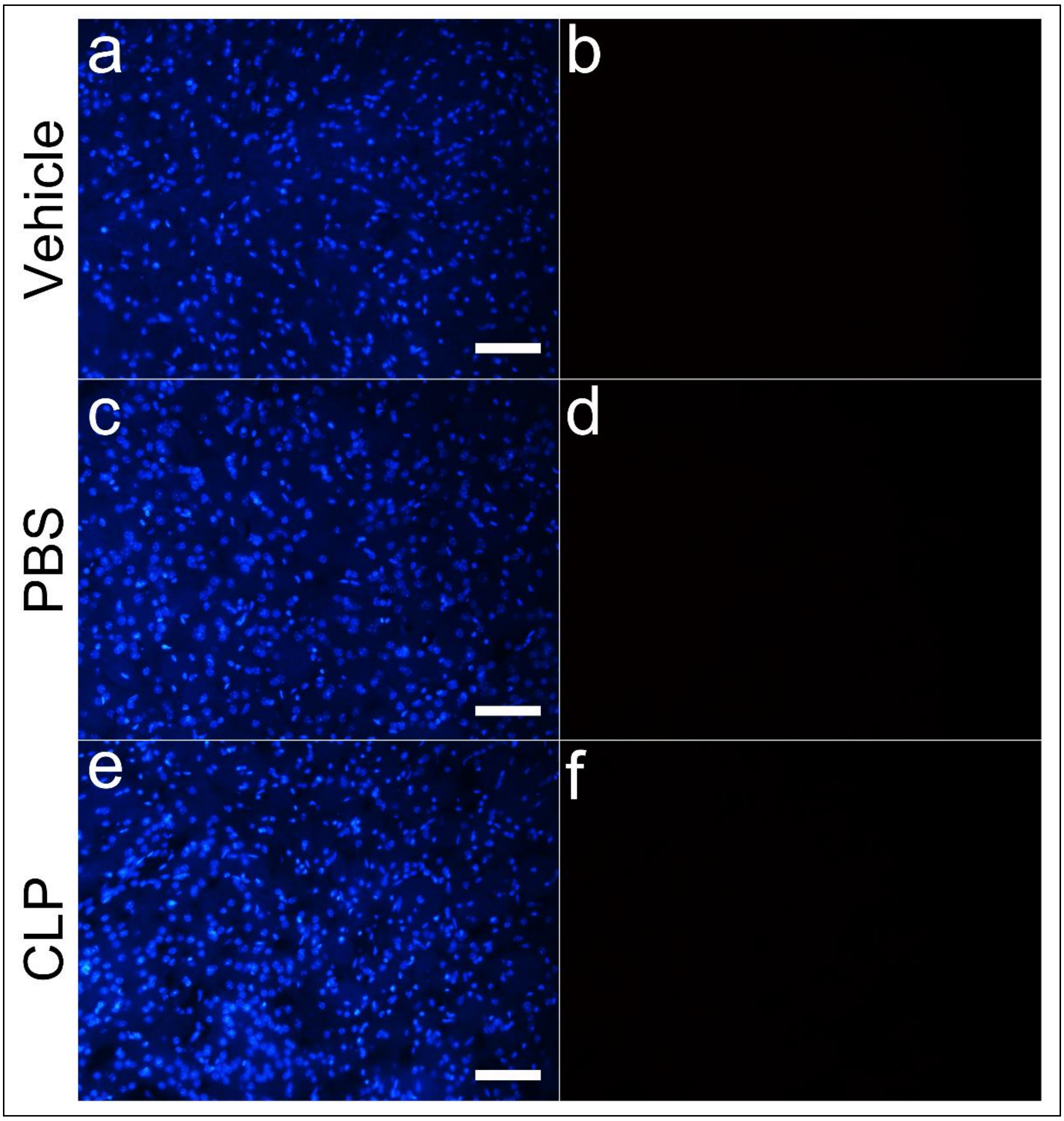

Supple 4. Negative control of CD31 immunostaining (no primary antibody of CD31). Presentation of DAPI stained nuclei (**a, c, e,**) and CD31 negative immunostaining (**b, d, f**) in the peri-infarct region of the striatum of Vehicle, PBS, and CLP treated group. Scar bar=50 μm.
